# Supplementary material for: mDrop-Seq: Massively Parallel Single-Cell RNA-Seq of Saccharomyces cerevisiae and Candida albicans
Source: Vaccines (Basel). 2021 Dec 27;10(1):30. doi: 10.3390/vaccines10010030 (PMC8779198; doi:10.3390/vaccines10010030)
Supplement: Supplementary file 1 [file vaccines-10-00030-s001.zip › vaccines-1453234-supplementary.pdf]

Supplementary Material

Optimization of mDrop-seq for *Saccharomyces cerevisiae*

mDrop-seq requires cell lysis after single cells are encapsulated in droplets. We developed targeted in-drop lysis protocols for each species that consist of cocktails of zymolyase, sarkosyl and incubation at 37 °C. Sarkosyl is a strong detergent used for cell lysis [1]; zymolyase is an enzyme mixture with optimal activity at 37 °C, commonly used to digest yeast cell wall. We note that zymolyase or sarkosyl alone were insufficient to obtain total lysis of yeast cells in drops. Because droplet stability is influenced by constituent fluids and flow parameters, we optimized the Droplet Yeast Lysis Buffer (DYLB) composition with oil-surfactant mix, droplet size and flow rates for each yeast species. Monodisperse droplets of 75 μm diameter that are stable under flow and thermal incubation were used for mDrop-seq experiments. The scRNA-seq workflow in yeasts in microfluidic drops is summarized in Figure 1A, left.

Batch correction in *Saccharomyces cerevisiae* replicates

The control experiment was repeated with 15 and 20 min incubation times for lysis, labeled SC_15min_Rep2 and SC_20min_Rep2, respectively. For both datasets, we obtained slightly lower numbers of cells, and comparable numbers of genes and UMI per cell within the datasets (Table S1 and Figure S2A). Overall, the two lysis times in replicate 2 yielded comparable data with Pearson correlation of 0.98.

The two replicates, SC_XXmin_Rep1 and SC_XXmin_rep2 (Pearson correlation = 0.86) show overlapping but slightly different sets of differentially expressed genes and some differences in their expression profiles. Several genes that are more highly expressed in replicate 2 included *HSP12*, *HSP42*, *DDR2*, and *NCE103* (Figure S2B), many of which involve stress response. Figure S2C shows the 15 highest expressed genes in replicate 2, with *HSP82*, *HSP12*, and *HOR7* being among the top 4 genes expressed. This, along with SC_20min_Rep2 having slightly higher genes and UMI detected compared to SC_15min_Rep2, may indicate that this replicate underwent longer lysis incubation or otherwise experienced stress.

A PC plot combining the two replicates with two lysis incubation times each into a single dataset of 20,548 cells (Figure S2D) shows clear separation between SC_XXmin_Rep1 and SC_XXmin_Rep2. These replicates were performed on different days and may have sampled *S. cerevisiae* cells from slightly different growth phases during culture. Nevertheless, we were able to adjust for these differences computationally (Figure S2E) using anchor based integration in Seurat [2].

Because of the noted increase in stress response in the second replicate, we compared the second "stressed" control replicate as experiencing intermediate levels of heat shock related stress between the *S. cerevisiae* replicate 1 and the heat shock experiments. Indeed, when comparing expressions of, Figure S3C shows increased overall expression of *HSP12*, *HSP26*, and *SSA4* in replicate 2 or the intermediate "stressed" control, though lower than expression in the heat shock data (logFC = 1.99, 2.04, 2.29, respectively). Housekeeping genes show similar expression across control (SC_XX_Rep1), intermediate (SC_XX_Rep2) and heat shock data (Figure S3D).

Analysis of cell cycle phases in *Saccharomyces cerevisiae*

In an unstimulated dataset sampling continuously cycling cells, one may expect to find variation attributable to cycling cells. Using cell-cycle gene lists provided in by Spellman *et al.* [3], we assigned a unique G1, S, or G2/M stage of the cell cycle to each *S. cerevisiae* cell that passed our quality filter. Due to low transcript counts inherent in yeasts, we were able to assign G1, S and G2M phases to only a subset of cells in each experiment (Table S2). The remaining cells were designated as 'not assigned' or NA. Cells in the same stage of cell-cycle largely grouped together in UMAP space (Figure S1D). Cell-cycle genes in the

S and G2M phases appear enriched in the upper left clusters (clusters 0 and 3 in Figure 1C, respectively) while G1 and additional G2M cells are spread across the lower clusters (clusters 1, 2, and 5 in Figure 1C). All cell-cycle phases are represented in the upper right cluster (corresponding to cluster 4 in Figure 1C). Figure S1E shows six histone genes enriched in cells assigned to S phase, as expected. After regressing out cell cycle effects, cluster 2 in Figure S1F shows increased expression of stress response genes, *DDR2, NCE103, HSP12, TMA10* (shown as feature plots in Figure S1G), likely induced during cell lysis. Figure S1H shows expression of genes *AIM44* and *PIR1* associated with GPI-anchored cell wall proteins that cluster together, independently of the cell cycle.

Analysis of potentially stressed *Candida albicans* shows differential expression

Lysis replicates of *C. albicans,* CA_20min_Rep1 and CA_25min_Rep1, showed heterogeneity both between and within experimental libraries. In this replicate, the 15 min lysis also produced a usable library, but was not included due to differences in total counts per cell with the other two time-points (Table S1). Gene cutoffs were determined for both 20 and 25 min lysis times at 370 and 250 respectively. A total of 4,006 cells were detected across the two lysis times. Lysis time of 20 min outperforms the other lysis times, with an average of 735 genes and 1,356 UMI detected. We combined the 20 and 25 min incubation datasets, CA_20min_Rep1 and CA_25min_Rep1 to construct a combined dataset with lower and upper cutoffs of 250 and 2,000 genes per cell (Pearson correlation = 0.95). The top expressed genes in this dataset are shown in Figure S5A. Much like CA_15min_Rep2 and CA_20min_Rep2 in Figure 3C, integration [2] was performed prior to combining these datasets (Figure S5B).

We saw several distinct clusters (clusters 6-10) in Figure S3B that separated out from the rest of the cells. These clusters have a slightly higher average number of genes (left) and UMI or counts (right) detected (Figure S6). Cluster 6 shows differential expression of genes associated with cell wall stability, e.g., *FGR41*, *PGA38*, and *SCW11* (p_adj<1e-292; Figure S5C). *CHT3*, a chitinase gene, was also expressed in cluster 6 (Figure S5C). Transcription factors *STP4* and *ADR1*, encoding for zinc finger proteins and implicated in *C. albicans* virulence, [4,5] and *GNP1*, a transmembrane transporter of amino acids, were moderately expressed across clusters (Figure S5D). *OLE1*, involved in filamentation, exhibited high expression across all cells, shown in Figure S5D. Cluster 10 represents cells producing histones, with many histone genes serving as the most significant markers for this cluster (p_adj<5.11e-33; Figure S5E).

Analysis of cell cycle phases in *Candida albicans*

Cell cycle marker genes for *C. albicans,* determined through microarray analysis of synchronized cells, were obtained from the Candida Cell-Cycle Database [6]. Cells from the combined *C. albicans* lysis datasets were scored for G1, S and G2M markers and assigned to the cell cycle phase with the highest score (Figure S7A for replicate 1, Figure S7B for replicate 2). As seen before in *S. cerevisiae* cells, we were able to assign cell cycle phases unambiguously to only a subset of *C. albicans* cells. The remaining cells were designated as NA. The number and percentage of cells in the different cell cycle phases in each dataset are summarized in Table S2. We note that the cells marked as NA (gray) are more or less uniformly distributed across the UMAP plot.

Figures S7C, D show the *C. albicans* replicates 1 and 2 after the variation from cell cycle genes were regressed out. Regressing out any variation in expression due to the cycling genes can be useful when comparing experimental conditions where the effects may be small (e.g., environmental stimuli).

For the experiments where *C. albicans* cells were treated with fluconazole, we noted significant increase in the fraction of cells assigned to the S phase and decrease in the fraction of cells assigned to the G2M phase, compared to their controls. The fraction of cells in the G1 phase did not change much, by comparison. These observations are consistent for both replicates of fluconazole treatment. This is also qualitatively similar to *S. cerevisiae* cells that underwent heat shock treatment.

To verify that cell assignment to the S phase was not biased by high expression of histone genes, the expression levels for S phase markers were examined with and without the contribution of histone genes. Figures S9C, E show dot plots of expression levels for S phase marker genes for all assigned phases of the cell-cycle (G1, S, G2M and NA) in the fluconazole datasets, CA_Fluconazole_Rep1 and CA_Fluconazole_Rep2. The average expression levels of all S phase genes in the cells assigned to the S phase were higher than the expression in the remaining cells. Even when the histone genes were excluded from the S phase marker list (Figures S9D, F), cells assigned to the S phase showed higher expression than G1 and G2M phases. This indicates that high S phase assignment among the *C. albicans* cells was not driven by the high expression of histone genes alone.

Supplemental References

[1] S. Frankel, R. Sohn, and L. Leinwand, *The use of sarkosyl in generating soluble protein after bacterial expression*; *Proc. Natl. Acad. Sci.*, : Publisher Location, Country 1991; Volume 88, pp. 1192–1196, https://doi.org/10.1073/pnas.88.4.1192.

[2] T. Stuart *et al.*, *Comprehensive Integration of Single-Cell Data; Cell*, 2019, Volume 177, pp. 1888-1902.e21, https://doi.org/10.1016/j.cell.2019.05.031.

[3] P. T. Spellman *et al.*, *Comprehensive identification of cell cycle-regulated genes of the yeast Saccharomyces cerevisiae by microarray hybridization*; *Mol. Biol. Cell*, 1998, Volume 9, pp. 3273–3297, https://doi.org/10.1091/mbc.9.12.3273.

[4] J. F. Muñoz *et al.*, *Coordinated host-pathogen transcriptional dynamics revealed using sorted subpopulations and single macrophages infected with Candida albicans*; *Nat. Commun.*, 2019, Volume 10, p. 1607, https://doi.org/10.1038/s41467-019-09599-8.

[5] L. Issi *et al.*, *Zinc Cluster Transcription Factors Alter Virulence in Candida albicans*; *Genetics*, 2017, Volume 205, pp. 559–576, https://doi.org/10.1534/genetics.116.195024.

[6] P. Côte, H. Herve, and M. Whiteway, *Transcriptional Analysis of the Candida albicans Cell Cycle*; *Mol. Biol. Cell*, 2009, Volume 20, pp. 3363–3373, https://doi.org/10.1091/mbc.E09.

Supplemental Tables and Figures

Table S1. Summary of *S. cerevisiae* and *C. albicans* datasets showing average read counts, genes, and UMI detected per cell for mDrop-seq experiments. The average reads and counts for the species-mixing experiments are reported after filtering out misaligned genes.

Library	Raw Counts	Cells Detected	Average Counts per cell	Average Genes per cell	Median Genes per cell	Average UMI per cell	Median UMI per cell
SC_15min_Rep1	227,973,901	6,620	34,437	474	417	781	639
SC_20min_Rep1	137,063,719	5,392	25,419	402	347	626	502
SC_15min_Rep2	108,347,980	4,350	24,907	380	334	503	424
SC_20min_Rep2	58,362,030	4,740	12,312	404	365	529	460
SC_Heat Shock_Rep1	65,393,958	7,483	8,739	434	377	721	590
SC_Heat Shock_Rep2	54,662,686	6,524	8,740	587	515	1,053	866
Species Mix- SC - 7%	88,687,370	1,495	21,288	544	487	1,108	911
Species Mix- CA - 7%	88,687,370	2,671	21,288	193	137	422	348
Species Mix - SC - 15%	88,953,431	4,901	10,962	578	529	1,173	981
Species Mix - CA - 15%	88,953,431	3,214	10,961	797	717	1,565	1,276
CA_15min_Rep1	30,400,506	1,215	25,021	451	376	685	504
CA_20min_Rep1	93,129,841	1,693	55,008	837	706	1,660	1,223
CA_25min_Rep1	128,016,172	2,364	54,152	573	494	1,029	783
CA_15min_Rep2	96,778,937	6,452	7,132	508	549	836	610
CA_20min_Rep2	129,649,186	3,862	15,021	611	561	1,132	976
CA_Fluconazole-ctrl_Rep1	154,646,838	5,952	25,982	563	510	944	784
CA_Fluconazole-1.5hr_Rep1	122,638,009	4,924	24,906	893	816	1,948	1586
CA_Fluconazole-3hr_Rep1	111,642,039	4,627	24,128	953	922	2,130	1,882
CA_Fluconazole-ctrl_Rep2	95,442,703	4,227	10,918	351	324	527	456
CA_Fluconazole-1.5hr_Rep2	115,687,150	3,687	12,978	967	918	2,152	1,863
CA_Fluconazole-3hr_Rep2	96,686,038	3,028	11,989	935	896	1,909	1,689

Table S2. The number and percentage of cells in different cell cycle phases across the different experimental replicates.

Dataset	G1 Cells	G1 (%)	S Cells	S (%)	G2M Cells	G2M (%)	NA Cells	NA (%)
SC_Rep1	1,194	15.8	3,435	45.4	2,938	38.8	1,523	16.7
SC_Rep2	2,453	27.5	3,615	40.5	2,862	32	3,082	25.7
SC_Heat Shock _Rep1	1,016	18.4	3,770	68.2	738	13.4	1,369	19.9
SC_Heat Shock _Rep2	831	15.3	3,636	66.8	979	18	1,078	16.5
CA_Rep1	268	14.6	862	47.1	702	38.3	2,174	54.3
CA_Rep2	671	11.4	3,282	55.8	1,932	32.8	4,429	42.9
CA_Fluconazole-ctrl_Rep1	585	25.3	702	30.3	1,029	44.4	3,636	61.1
CA_Fluconazole-1.5hr_Rep1	370	11.7	2,257	71.7	522	16.6	1,775	36
CA_Fluconazole-3hr_Rep1	603	22.2	1,325	48.9	782	28.9	1,917	41.4
CA_Fluconazole-ctrl_Rep2	351	17.9	756	38.6	854	43.5	2,266	53.6
CA_Fluconazole-1.5hr_Rep2	233	10.2	1,344	58.8	708	31	1,402	38
CA_Fluconazole-3hr_Rep2	273	16.6	927	56.3	447	27.1	1,381	45.6

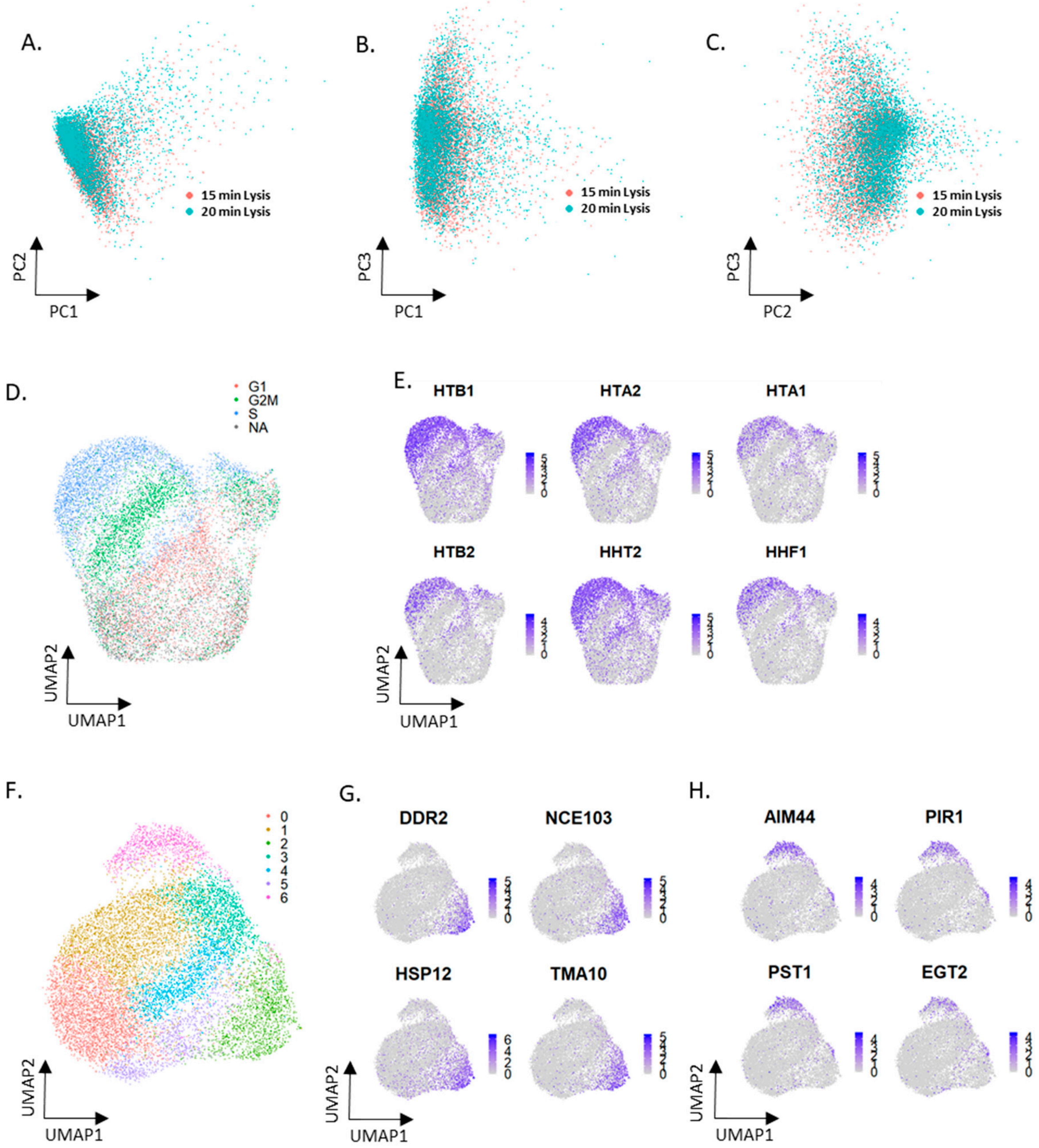

Figure S1. Analysis of 12,012 *S. cerevisiae* cells, replicate 1. A-C) Plots of PCs 1-3 displaying the overlapping datasets. D) UMAP of *S. cerevisiae* labeled by cell cycle stages. Cells that could not be assigned to a cell cycle phase are marked as NA. E) Feature plots of histone genes enriched in S phase cells. F) UMAP plots of cells after cell-cycle regression, labeled by clusters obtained by unsupervised clustering. G) Stress response genes *DDR2*, *NCE103*, *HSP12*, and *TMA10* form a separate cluster (cluster 2) after cell cycle regression. H) Feature plots of *AIM44*, *PIR1*, *PST1, and EGT2* for cell wall and budding marker expression that cluster separately (cluster 5) after cell cycle regression.

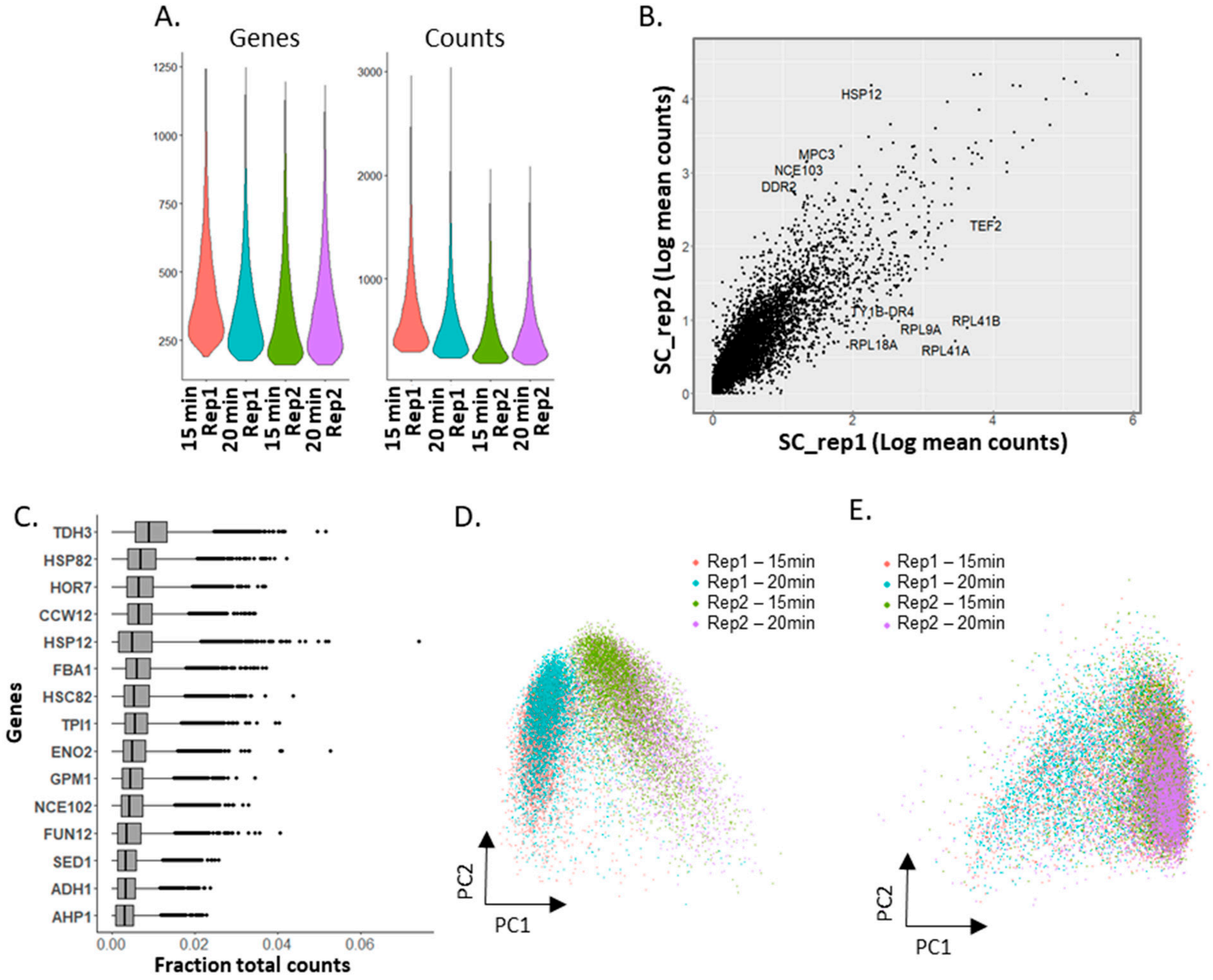

Figure S2. Comparison of replicates for different *S. cerevisiae* lysis incubation time experiments**.** A) The number of genes and UMI detected in 4 datasets. B) Plot of the average expression of genes in 22,491 *S. cerevisiae* cells from the control replicates. C) Top 15 genes expressed in the second replicate dataset. D-E) Plots of PC 1 and 2 displaying the four datasets without (D) and with (E) batch correction.

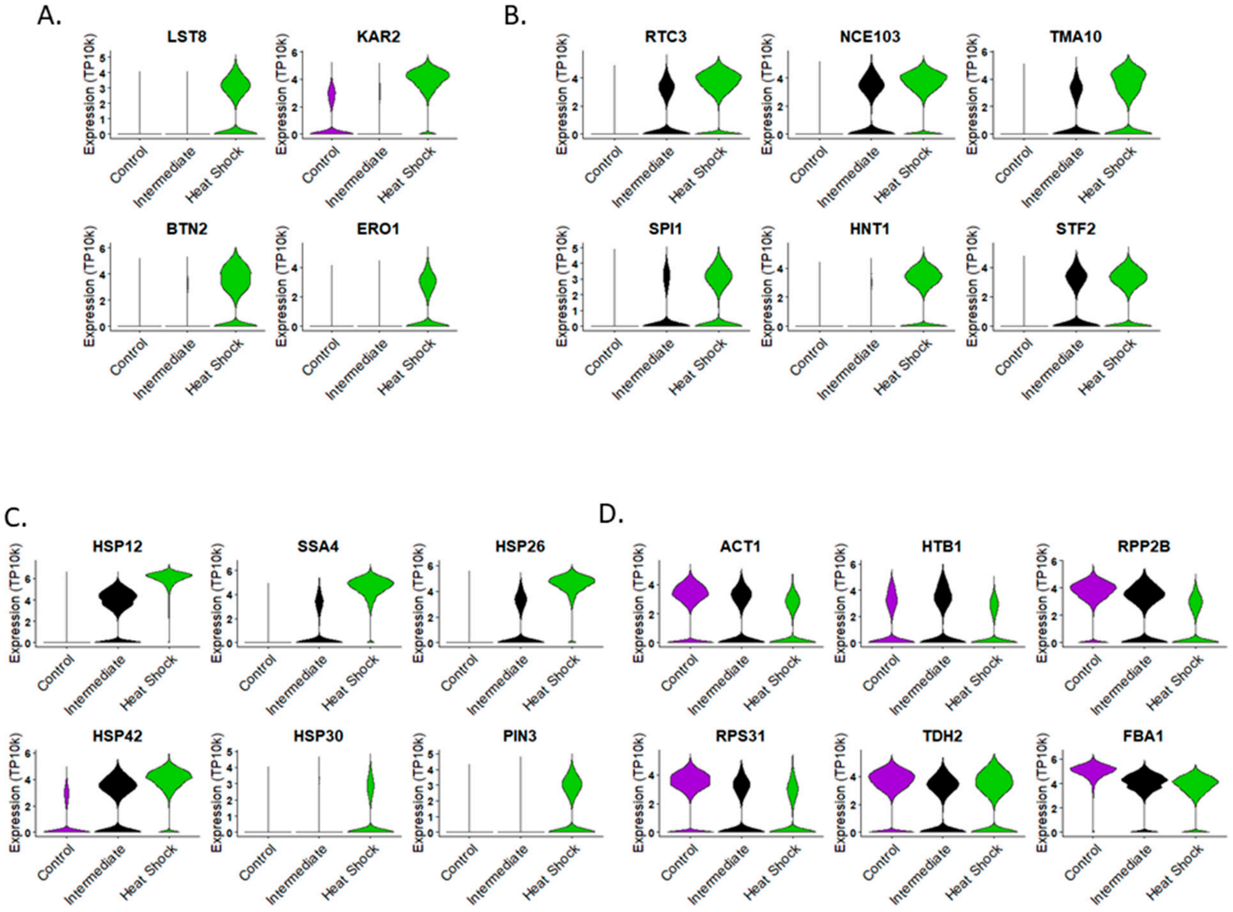

Figure S3. Analysis of 35,109 *S. cerevisiae* cells including 14,007 cells after heat shock and 21,102 cells as control. A) Four genes for DNA replication stress show significant upregulation during heat shock when compared to the replicate 1 control. B) Several genes involved in protein transport through the ER and Golgi show elevated expression during heat shock. C) Violin plots displaying heat shock genes that are progressively elevated in the second control replicate and heat shock experiments. D) Violin plots displaying housekeeping genes (actin, histones, ribosomes, and glycolysis) that appear in both heat shock and control replicate 2 data.

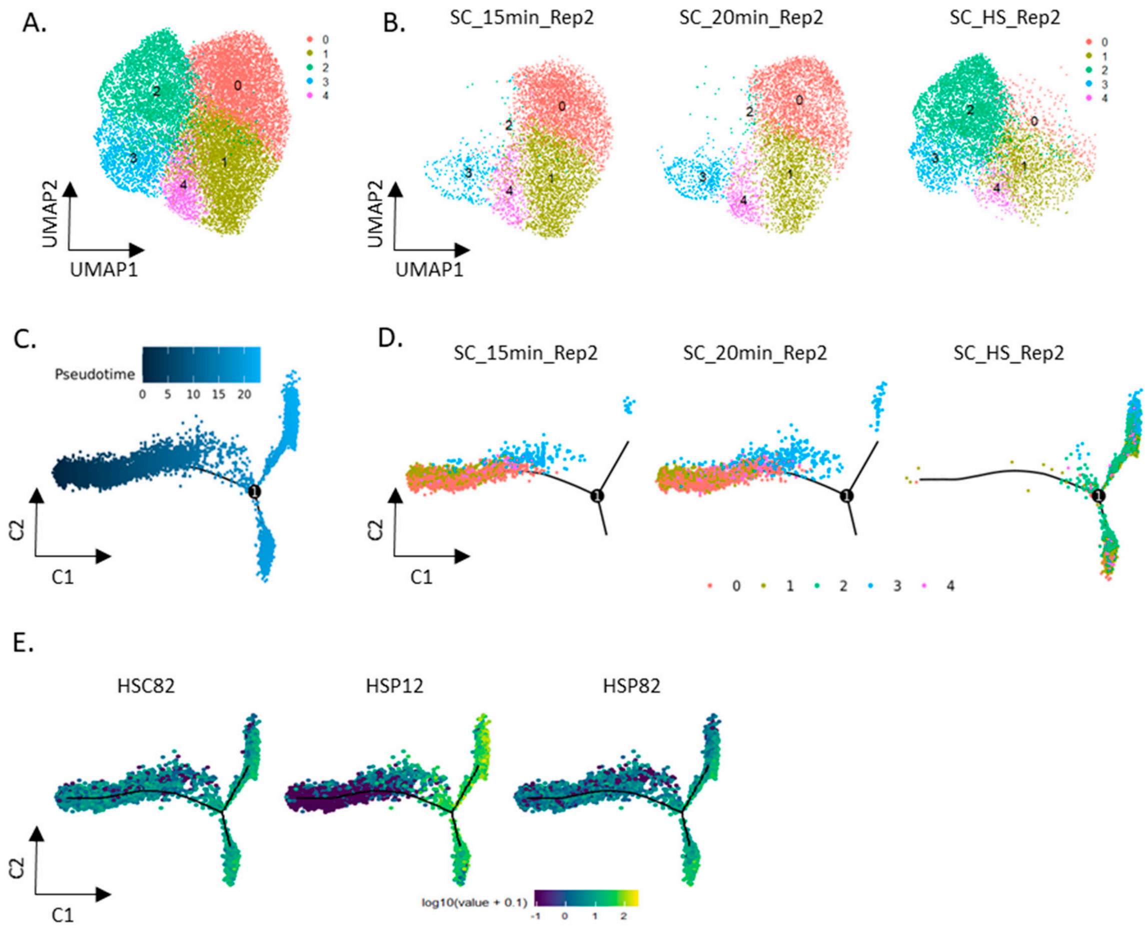

Figure S4. Clustering and pseudo-time analysis of *S. cerevisiae* cells. A) UMAP plot of unsupervised clustering on integrated combined control (15 & 20 min lysis) and heat shocked *S. cerevisiae*, replicate 2 after the cell cycle effects are regressed out. Colors represent cell clusters also shown in Figure 2H. B) UMAP plots displaying the membership of cells from the controls (15 min-left; 20 min- middle) and heat shocked (right) *S. cerevisiae* cells, replicate 2. C) Inferred pseudo-time trajectory of the combined dataset. Color bar indicates pseudo-time. D) Inferred trajectories split by control (15 min-left; 20 min- middle) and heat shocked (right) datasets shown in Figure 2H. Colors represent cell clusters shown in Figure 2H. E) Expression of heat shock genes *HSC82*, *HSP12* and *HSP82* along the pseudo-time trajectory. Color bar indicates scaled expression level.

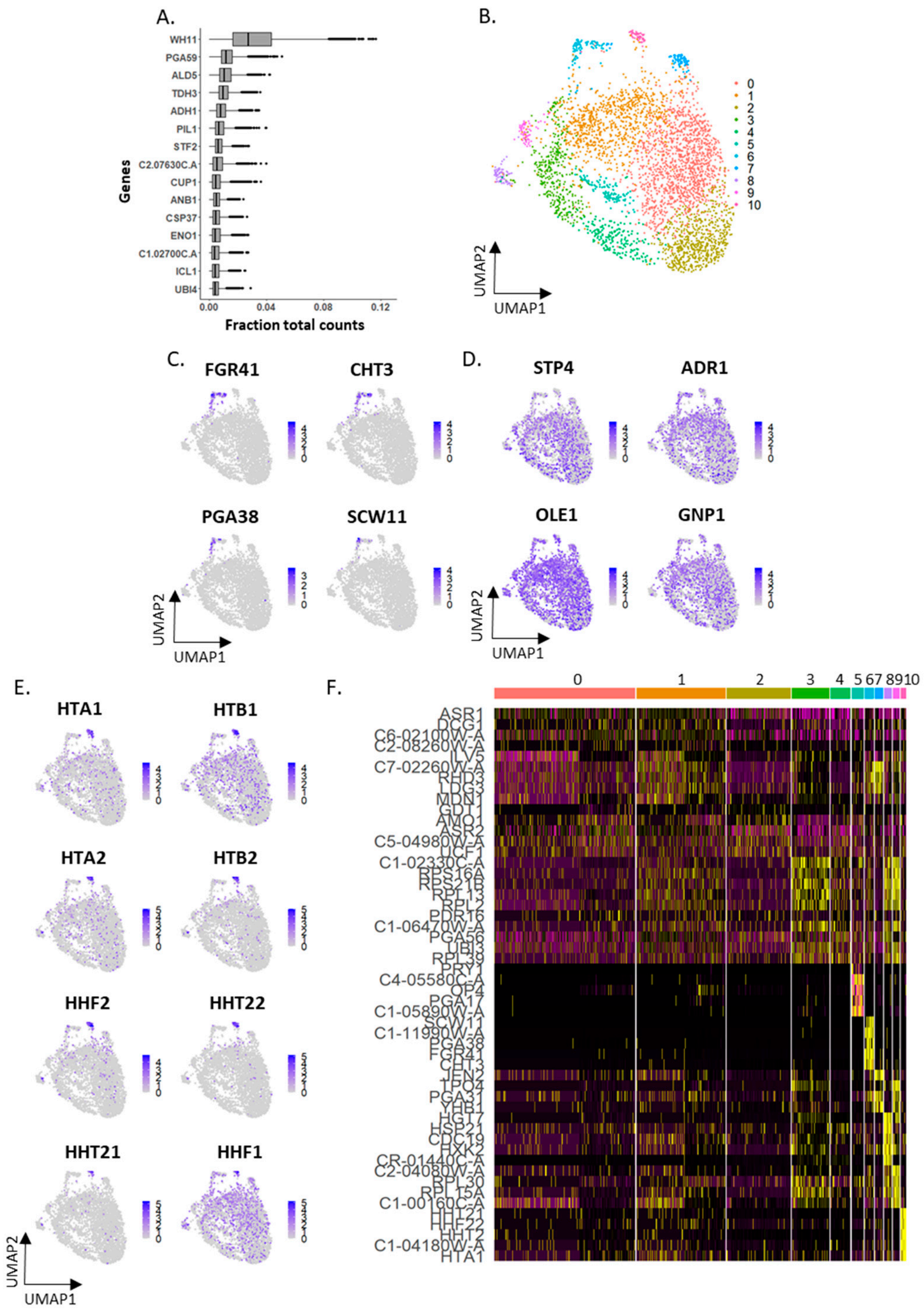

Figure S5. mDrop-seq of 4,006 *Candida albicans* cells. A) Boxplot of the 15 highest expression genes by the fraction of total counts in *C. albicans* data. B) UMAP of clustering analysis of *C. albicans* cells after batch correction. C) Feature plots displaying cell wall genes that represent markers for cluster 6. D) Feature plots of transcription factors, fatty acid biosynthesis, and hyphal formation genes implicated in *C. albicans* virulence. E) Feature plots displaying histone tail genes that mark cluster 10. F) Heatmap displaying expressions of the top marker genes for each cluster shown in B.

A.

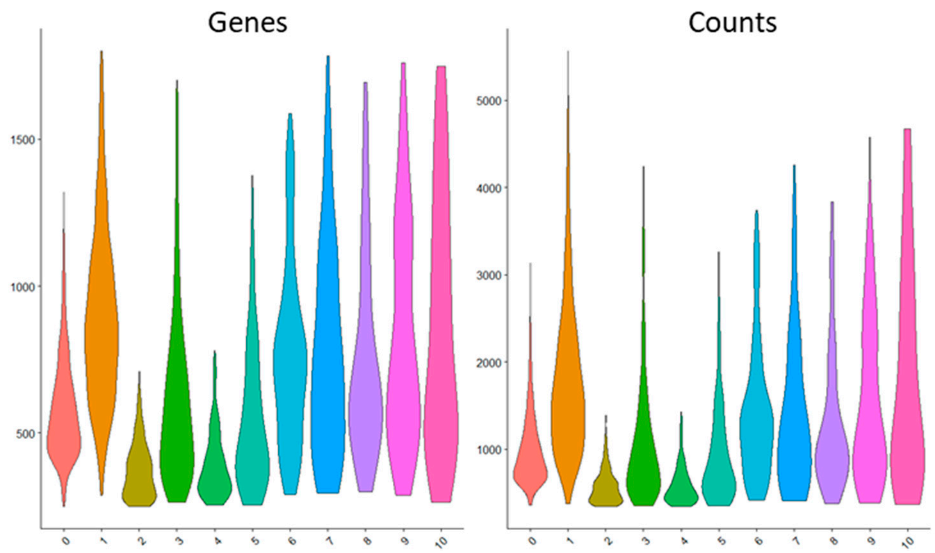

Figure S6. Gene and UMI Counts per cluster for *C. albicans* cells, replicate 1. A) *C. albicans* expression levels across clusters in Figure S5B. Clusters 6-10 containing histone genes, glycolysis genes, and cell wall genes, show slightly higher numbers of genes or UMI detected compared to other clusters.

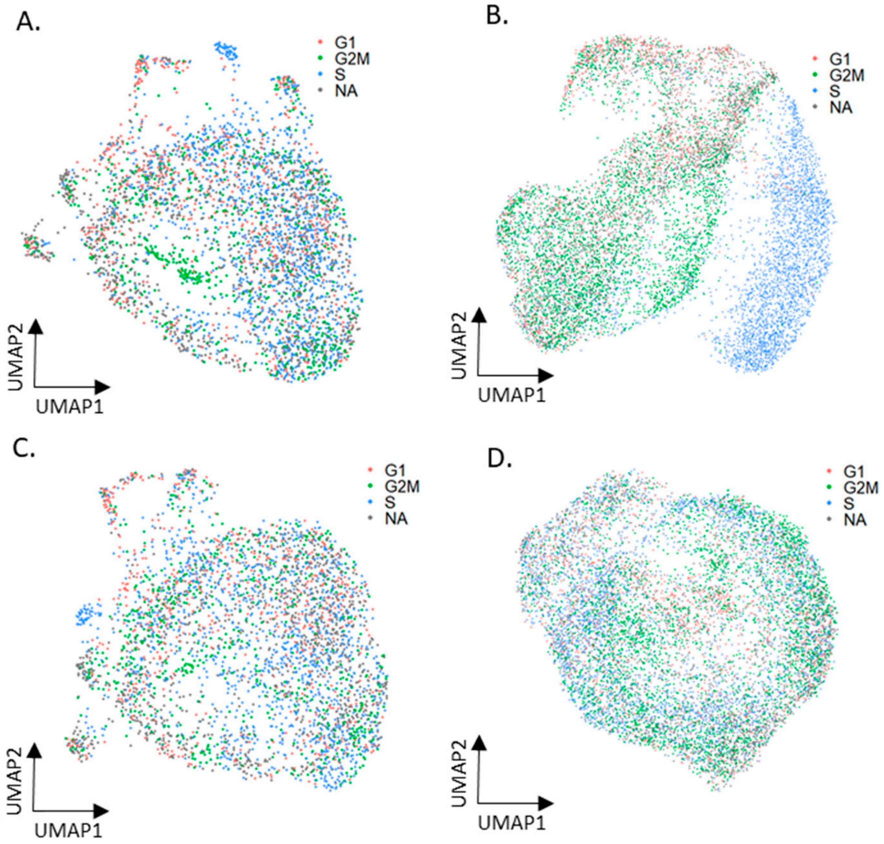

Figure S7. Analysis of cell cycle genes in *C. albicans* replicates. A) UMAP plot of replicate 1 (Figure S5B) with cells marked for the G1, G2M and S phases of the cell cycle. B) UMAP plot of replicate 2 (Figure 3C) with cells marked for the G1, G2M and S phases of the cell cycle. C) UMAP plot of replicate 1 showing the integrated dataset after regressing out the variations from cell cycle. D) UMAP plot of replicate 2 after regressing out the variations attributed to cell cycle.

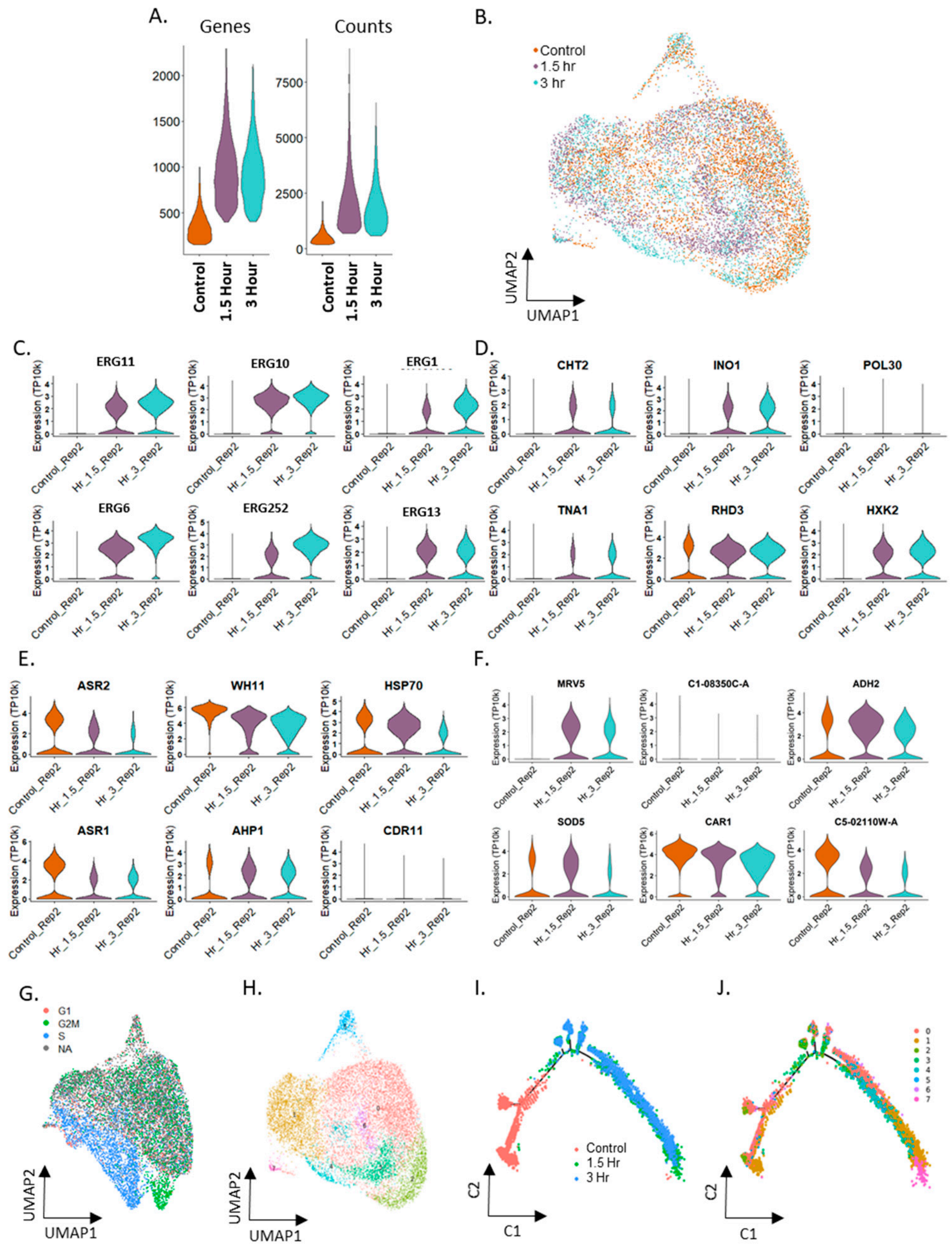

Figure S8. Fluconazole treatment of 10,942 *C. albicans* cells, replicate 2, profiled using mDrop-seq. A) Violin plots displaying the number of genes and UMI for each heat shock replicate. B) UMAP displaying the clustering patterns of the integrated control and fluconazole libraries. C-F) Violin plots of genes that show differences in expression between control and fluconazole treated datasets for C) ergosterol biosynthesis, D) genes that increases with time under fluconazole exposure, E) genes with decreasing expression under fluconazole exposure, F) genes with significant differential expression for the 1.5 hr time-point. G-H) UMAP plots of the integrated data in replicate 2, labeled by G) cell cycle, and H) unsupervised clusters after cell cycle regression. I, J) Pseudo-time trajectory of the replicate 2 inferred using Monocle. Colors indicate I) experimental time-points, and J) cell-type clusters shown in H.

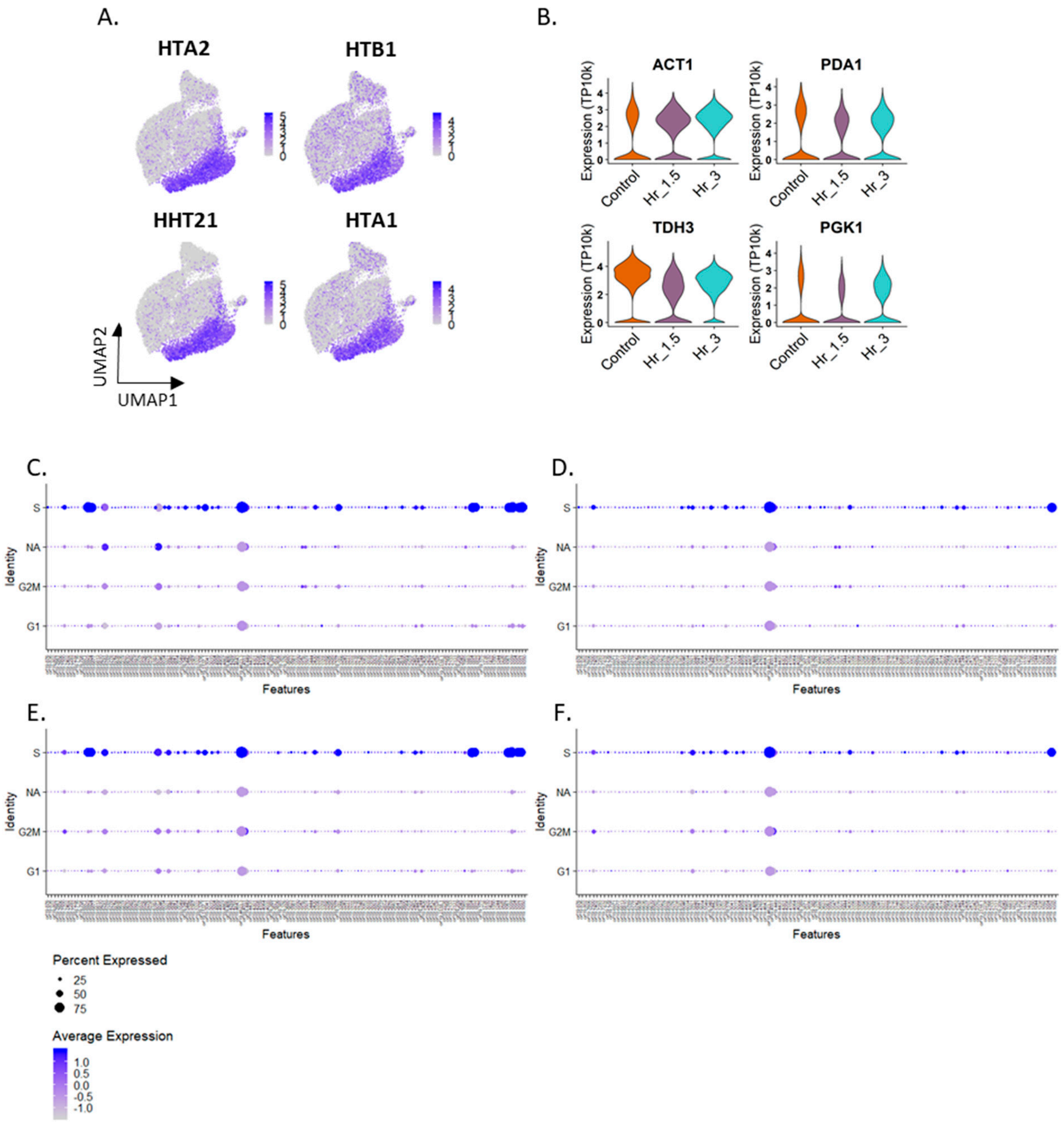

Figure S9. Cell cycle analysis of 15,503 control and fluconazole treated *C. albicans* cells, replicate 1. A) Feature plots displaying four histone tail genes across control and fluconazole exposed cells. B) Expression levels of housekeeping genes *ACT1, PDA1, TDH3* and *PGK1* in *C. albicans*, replicate 1. C-F) Dot plots of S phase markers C) with histone genes on replicate 1; D) without histone genes on replicate 1; E) with histone genes on replicate 2; F) without histone genes on replicate 2.

.

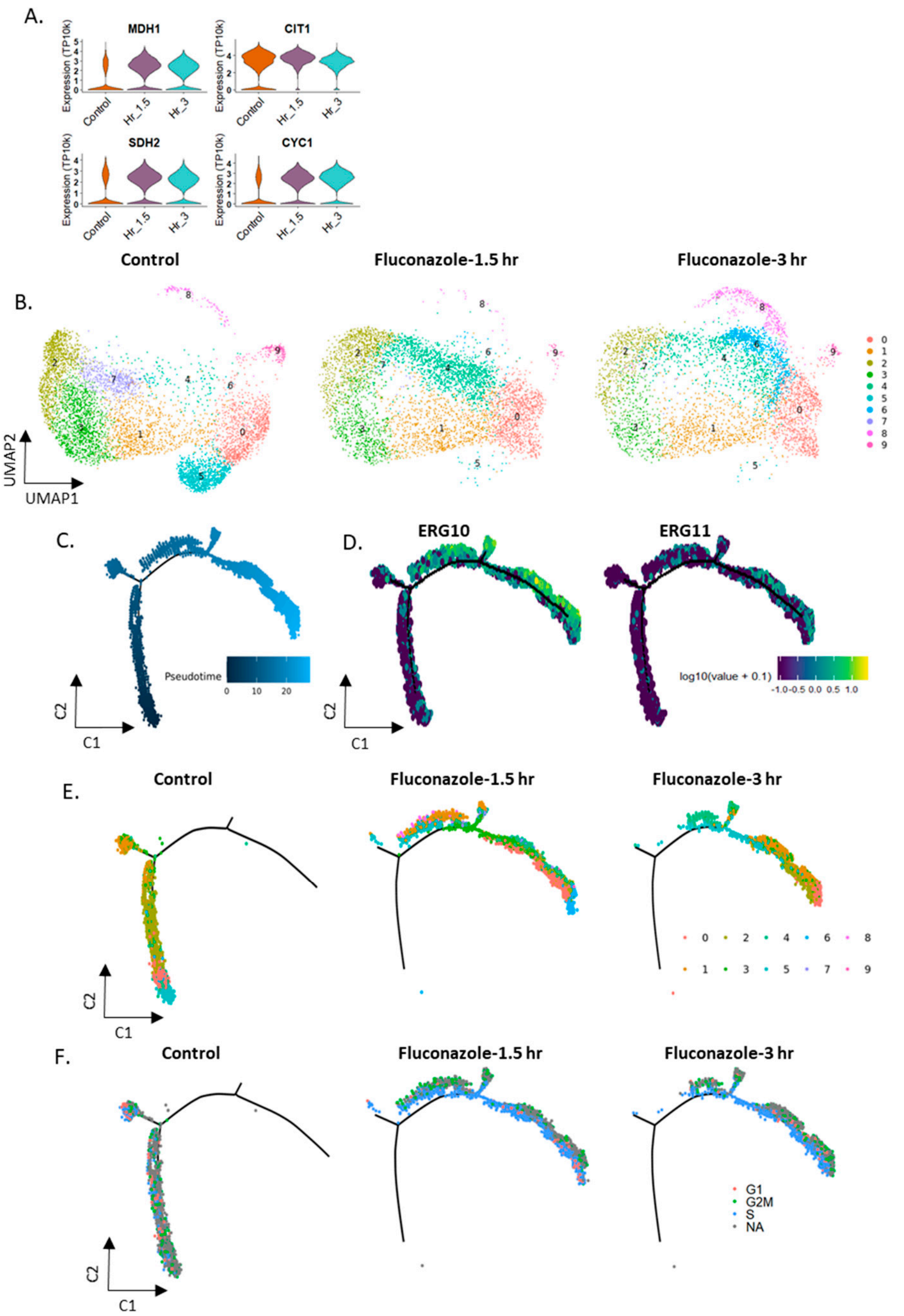

Figure S10. Fluconazole treatment of 15,503 *C. albicans*, replicate 1. A) Expression levels *of MDH1, CIT1, SDH2*, and *CYC1* genes that are differentially expressed in cluster 5 in Figure 4I. B) UMAP plots displaying the membership of cells from the control (left) and fluconazole (1.5 hr- middle; 3 hr- right) samples in the combined dataset shown in Figure 4I. C) Inferred trajectory of gene expression in *C. albicans*, replicate1 in response to fluconazole, with color bar indicating pseudo-time. D) Expression levels of ergosterol synthesis genes, ERG10 and ERG11 increase with pseudo-time. E-F) Inferred trajectories split by control (left) and fluconazole (1.5 hr- middle; 3 hr- right) datasets shown in Figure 4K. Colors represent E) cell clusters shown in Figure 4I, F) Cell cycle phases, G1, S, G2M and not assigned (NA).

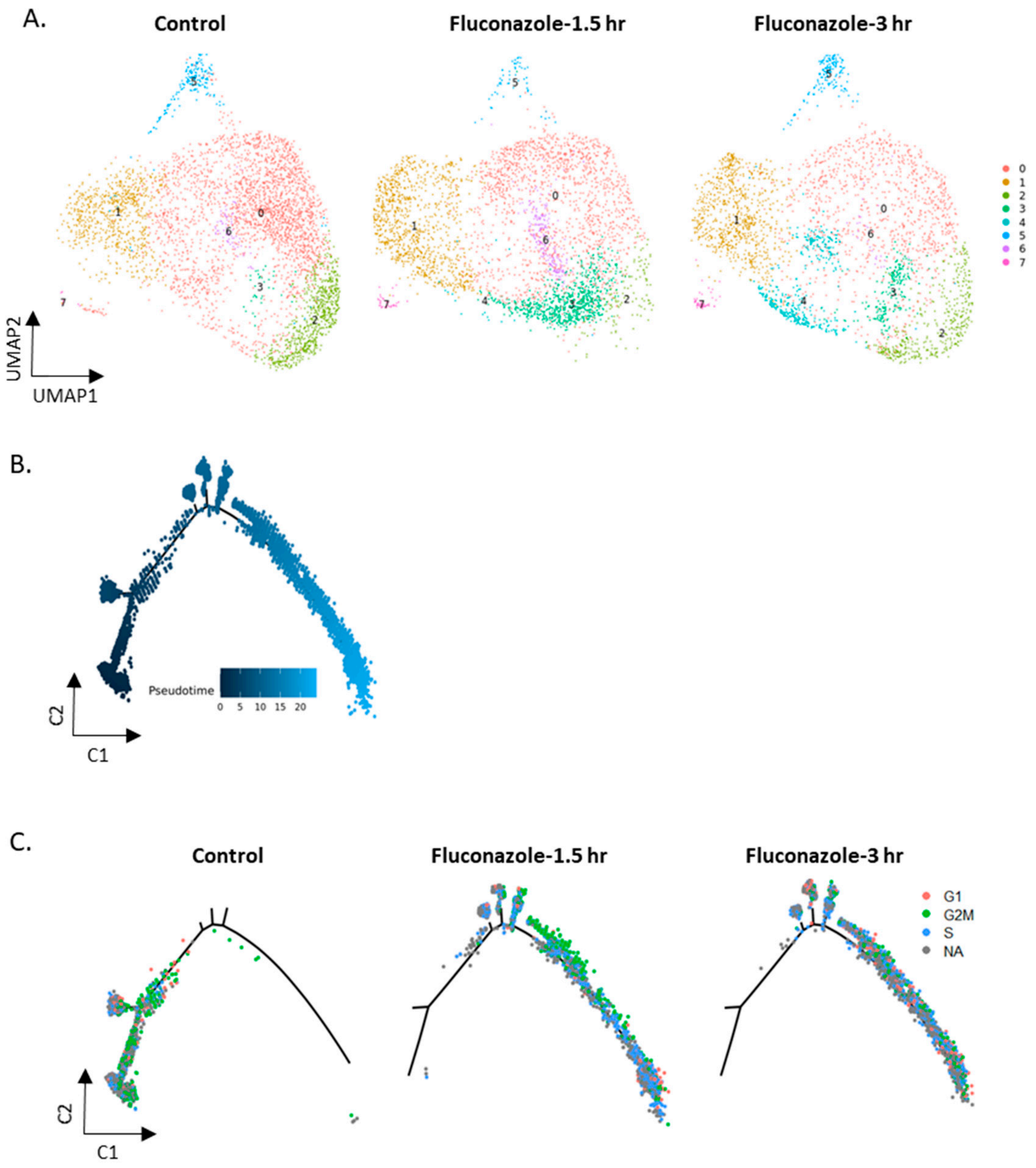

Figure S11. Fluconazole treatment of 10,942 *C. albicans*, replicate 2. A) UMAPs display the membership of cells from the control (left) and fluconazole (1.5 hr- middle; 3 hr- right) samples in Figure S10H. B) Inferred trajectory of gene expression in *C. albicans* in response to fluconazole, with color bar indicating pseudo-time. C) Trajectories split by control (left) and fluconazole (1.5 hr- middle; 3 hr- right) datasets in Figure S10H. Colors represent cell cycle phases, G1, S, G2M and not assigned (NA).

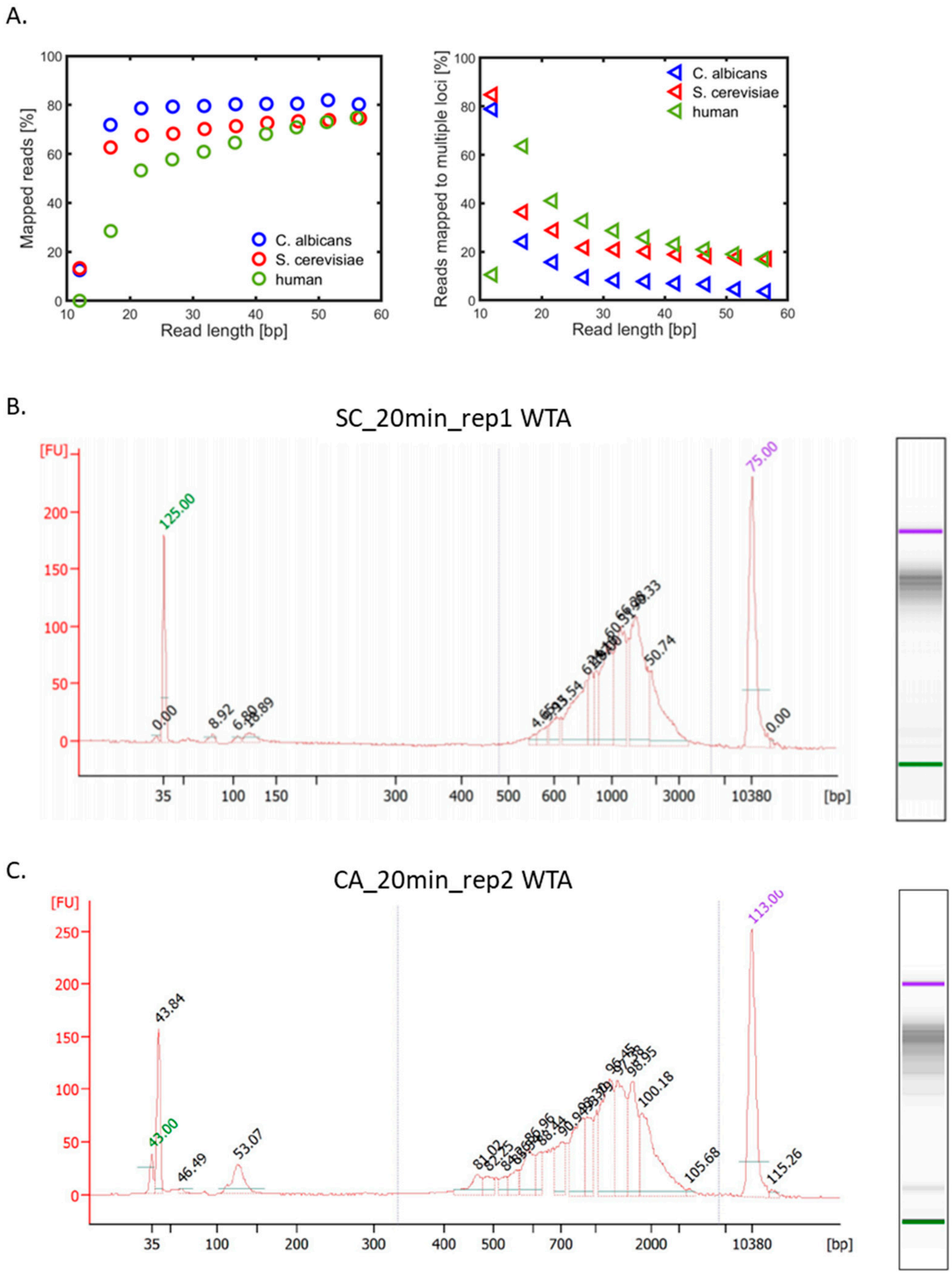

Figure S12. Quality control and sequencing considerations. A) Percent uniquely mapped reads (left) and reads mapped to multiple loci (right) as function of read length for *C. albicans*, *S. cerevisiae* and human genomes. B-C) Distribution of barcoded cDNA obtained from mDrop-seq experiments after Whole Transcriptome Amplification (WTA). B) *S. cerevisiae* and C) *C. albicans* shown in Agilent Bioanalyzer traces.
